# Prognostic Index for Nonsmall Cell Lung Cancer Based on Immune-Related Genes Expression

**DOI:** 10.1155/2022/4779811

**Published:** 2022-09-19

**Authors:** Ying Cao, Hongyu Zhu, Hailin Shen, Desen Liu, Zhenkai Li, Hailong Shang, Hongdi Du, Ying Wang, Juan Ye

**Affiliations:** ^1^Department of Radiotherapy, Suzhou Kowloon Hospital, Shanghai Jiaotong University School of Medicine, Suzhou 215028, China; ^2^Department of Radiotherapy, The Affiliated Suzhou Science & Technology Town Hospital of Nanjing Medical University, Suzhou 215153, China; ^3^Department of Radiology, Suzhou Kowloon Hospital, Shanghai Jiaotong University School of Medicine, Suzhou 215028, China; ^4^Department of Thoracic Surgery, Suzhou Kowloon Hospital, Shanghai Jiaotong University School of Medicine, Suzhou 215028, China

## Abstract

Immune system dysregulation is associated with tumor incidence and growth. Here, we established an RNA-based individualized immune signature associated with prognosis for nonsmall cell lung cancer (NSCLC) to guide adjuvant therapy. We downloaded publicly accessible data on RNA expression and clinical characteristics of NSCLC from the Cancer Genome Atlas (TCGA). From immune-related genes (IRGs) retrieved from the immunology database and analysis portal (ImmPort) database, we then screened differentially expressed immune-related genes (DEIRGs). Using overall survival (OS) as a clinical endpoint, we identified 26 prognostic DEIRGs via univariate and multivariate Cox regression analysis, and then developed a risk model based on these 26 IRGs with an area under the curve (AUC) of 0.701, and its predictive ability independent from other clinical factors. We also downloaded tumor immune infiltrate data and analyzed the correlations between lymphocytic infiltration with our risk scores, but found no significant association. Furthermore, we retrieved 86 differentially expressed transcription factors (TFs) to assess their regulatory relationships with the 26 prognostic DEIRGs. In summary, we developed a robust risk model to predict survival in patients with NSCLC, based on the expression of 26 IRGs. It provides novel predictive and therapeutic molecular targets.

## 1. Introduction

Worldwide, lung cancer is the most commonly diagnosed cancer (~11.6%) and accounts for the most cancer-related deaths (~18.4%). Due to the lack of characteristic early symptoms, almost 70 percent of lung cancer patients have developed the locally advanced or metastatic disease at the time of diagnosis. About 85% of lung cancers are NSCLC and have a poor prognosis; 5-year OS remains low (15%) across all stages [1]. Mortality and morbidity for NSCLC are similar, which indicates that its treatment is unsatisfactory, and has room for improvement.

Cancer immunotherapy is a personalized modality that leverages the immune system to combat tumors [2, 3]. It has shown long-term survival benefits; the 5-year CheckMate-003 follow-up study improved the 5-year survival rate of patients with advanced NSCLC from 4.9% to 16% [4]. In recent decades, advances in immunotherapy for numerous cancer types have become clinically available [5, 6]. However, as not all patients can benefit from these therapies, accurate screening for suitable candidates is the focus of much current research. Some biomarkers have proved useful in predicting patient survival and disease prognosis [7]. Programmed cell death protein-1 expression is the cornerstone for immunotherapy prediction. Combined with other indicators, it can help identify immunotherapy candidates.

Signatures based on IRGs have been explored in numerous studies to help stratify lung cancer patients' prognoses. Li et al. notably improved prognostic estimations among patients with nonsquamous NSCLC by establishing individualized immune signatures [8]; however, their findings were only applicable to patients with early-stage disease. Other studies have focused on lung adenocarcinoma [9, 10] or lung squamous cell carcinoma [11]. However, studies of its application to NSCLC have been sparse.

To understand further IRGs' clinical roles in NSCLC, including their prognostic significance and possible applications as targets for therapy, we developed an individualized prognostic risk model for NSCLC that relies on IRG transcription expression levels.

## 2. Materials and Methods

### 2.1. Data Acquisition from Public Databases

We downloaded data on NSCLC samples for transcriptome RNA sequencing from the TCGA portal database, including data for both adjacent nontumor lung tissues (*n* = 108) and tumor tissues (*n* = 1037) (all data has been normalized by fragments per kilobase per million), as well as clinical data including age, gender, and pathological TNM stage and OS from the patients who provided the samples. OS was used as the primary endpoint and defined as the time from diagnosis to death. The patients were censored if the date of death was unknown. We also downloaded the IRG list from ImmPort [12]. The ImmPort database provides timely and precise immunology data updates and offers an IRGs list that can be used for cancer research. The genes on the list were actively involved in immune activity.

### 2.2. Differential Gene Expression Analysis and Survival Analysis

We evaluated gene transcription data between adjacent nontumor and tumor samples to select differentially expressed genes involved in NSCLC onset, using the Wilcoxon test (R software limma package), setting a log2 |fold change|>1 and a false discovery rate (FDR) <0.01 as cutoff values. DEIRGs were extracted from these differentially expressed genes. We used analyses of functional enrichment that included the Kyoto Encyclopedia of Genes and Genomes (KEGG) and Gene Ontology (GO), via the Bioconductor package “clusterProfiler,” and visualized via “ggplot2.” The terms with FDR<0.05 were considered significantly enriched. OS was selected as the primary endpoint. Prognostic DEIRGs were then selected through univariate Cox analysis, using the R software survival package (*P* < 0.05). These prognostic DEIRGs were also subjected to functional enrichment analyses.

### 2.3. Constructing the Immune-Related Gene Prognostic Index (IRGPI-)-Based Risk Model

We performed multivariate Cox regression analysis on prognostic DEIRGs that were identified as significant in univariate Cox analysis. All independent prognostic indicators from the multivariate analysis were used for the IRGPI-based risk model. We calculated risk scores according to each IRG's expression level multiplied by the coefficient from Cox regression. We classified patients whose scores were below and above the risk score median value as low and high risk, respectively; differences were evaluated via the log-rank test. We used Kaplan–Meier survival curves to evaluate the differences between the two groups. The AUC of the survival receiver operating characteristic (ROC) curve was constructed to evaluate the model's performance using the survival ROC package in R software [13]. We evaluated OS correlation to clinical pathologic factors (gender, age, and pathological stage) and risk scores via univariate and multivariate Cox analyses.

### 2.4. Construction of a Transcription Factor Regulatory Network

We then explored the regulatory mechanisms of the IRGs included in the risk model. As TFs regulate gene expression, we wished to know the mechanisms through which the TFs operated. We downloaded information on 318 TFs from the Cistrome Cancer database [14]. TFs that showed differential expression were extracted to construct the molecular regulatory network with prognostic DEIRGs included in our risk model. Correlation coefficients >0.3 and *P* < 0.001 were regarded as significantly associated.

### 2.5. Estimating the Correlation of IRGPI and Tumor-Infiltrating Immune Cells

As immune cells are recognized as the main tumor immune microenvironment (TIME) portion, we downloaded immune infiltrate data of patients with NSCLC from the Tumor Immune Estimation Resource (TIMER) online database, which estimates the abundance of six types of immune cells, including B cells, CD4^+^ T cells, macrophages, dendritic cells, CD8^+^ T cells, and neutrophils [15]. The correlation of tumor-infiltrating immune cells with IRGPI was assessed by the Pearson correlation coefficient test.

### 2.6. Association between Model and Clinical Variables

We evaluated how our risk model is associated with gender, age, and pathological stage. As age is a continuous variable, we used the median as a cutoff. Pathological stages were described as dichotomous categorical variables (stages I–II vs III–IV; T1–2 vs T3–4; N0 vs N1–3; and M0 vs M1). Statistical comparisons of gene expression for two groups were evaluated by Student's *t*-test. R (version 3.6.1; https://www.r-project.org/) was used for all statistical analyses. *P* < 0.05 was considered significant unless specified otherwise.

## 3. Results

### 3.1. Construction of Differentially Expressed and Survival-Associated IRGs

**Figure 1:**
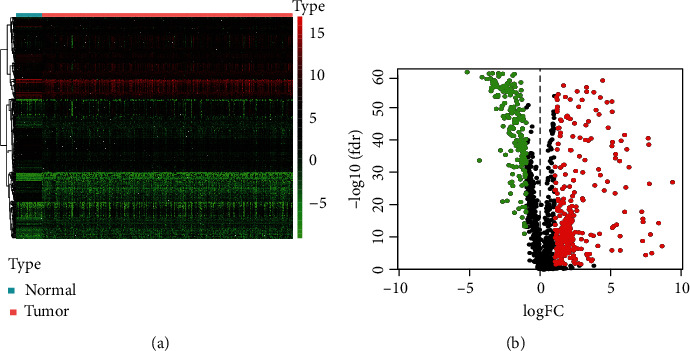
Identification of differentially expressed immune-related genes (IRGs). (a) IRGs heat map; the spectrum (green to red) shows gene expression from low to high. (b) IRGs volcano plot; red, green, and black points indicate the IRGs that were significantly upregulated, downregulated, and nonsignificantly differentially expressed, respectively. FC: fold change; FDR: false discovery rate.

Of the 7,336 genes that were differentially expressed, 5,439 were upregulated, and 1,897 were downregulated on the tumor samples. Of the 529 extracted DEIRGs, 333 genes were upregulated, and 196 were downregulated (Figure 1, Supplementary Table 1).

**Figure 2:**
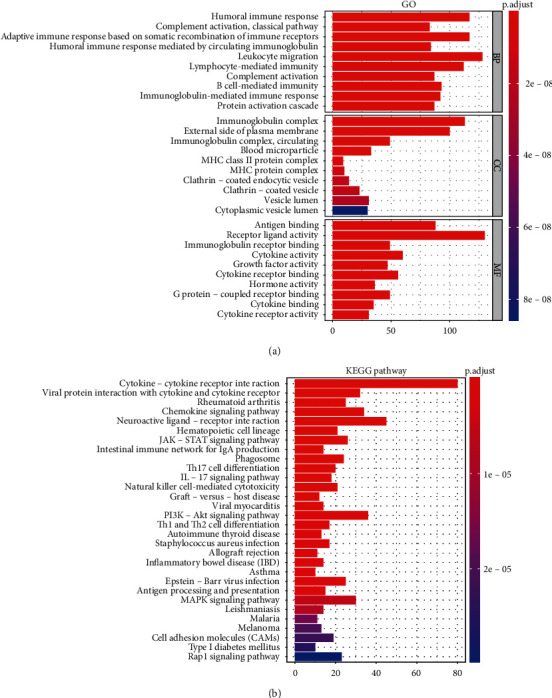
Functional enrichment analyses of differentially expressed immune-related genes. (a) Gene Ontology (GO) analysis. The *y*-axis indicates the GO terms, and the *x*-axis indicates the number of the genes. Only the top 10 GO terms of biological process (BP), cellular components (CC), and molecular functions (MF) are listed, respectively, in this figure. (b) Kyoto Encyclopedia of Genes and Genomes (KEGG) pathway analysis. The *y*-axis denotes the KEGG pathways, and the *x*-axis indicates the number of the genes. Spectrum (red to blue) indicates a significant level of enrichment from high to low.

GO functional enrichment analysis showed that the DEIRGs were significantly enriched for “humoral immune response” among biological processes, for “immunoglobulin complex” among cellular components, and for “antigen-binding” among molecular functions (Figure 2(a)); these GO terms are preferentially involved in immune functions. In the KEGG pathways, the above genes were significantly enriched in cytokine–cytokine receptor interactions (Figure 2(b)).

To develop prognostic signatures with potential clinical utility, we screened the 529 DEIRGs for correlations with clinical outcomes and found that 41 DEIRGs were significantly associated with OS. Enrichment analysis showed that these 41 prognostic DEIRGs were related to two major GO enrichment terms: extracellular region (GO:0005576) and growth factor activity (GO:0008083).

### 3.2. Construction of IRGPI-Based Risk Model

**Figure 3:**
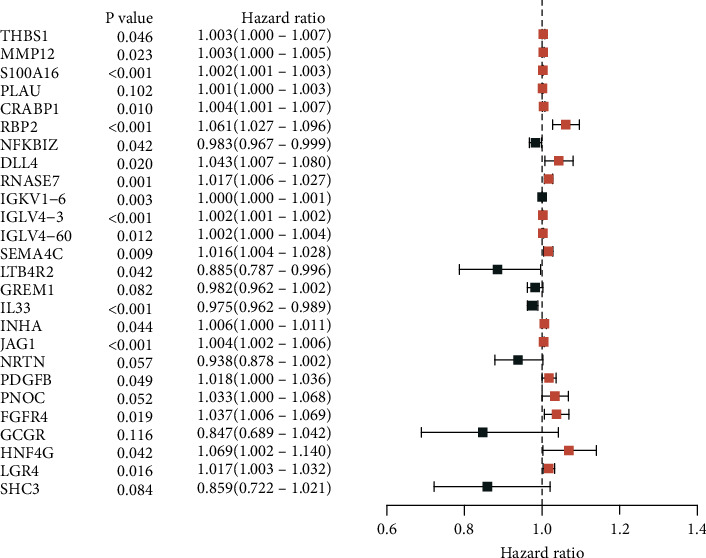
Forest plot demonstrating the multivariable Cox model results of each gene in the 26 immune-related genes-based risk model.

Of the 41 prognostic DEIRGs identified in univariate analysis for potential inclusion in the model, 26 remained as independent prognostic predictors after multivariate analysis (Figure 3). We calculated a risk score for each patient using their respective IRG expression levels × each IRG's Cox regression-determined coefficient, as shown below:

(1) $$\begin{array}{c} \text{Risk score}=\left(\text{THBS}1\times 0.00336909\right)+\left(\text{MMP}12\times 0.00265197\right)+\left(\text{S}100\text{A}16\times 0.00165915\right)+\left(\text{PLAU}\times 0.00113839\right)+\left(\text{CRABP}1\times 0.00398924\right)+\left(\text{RBP}2\times 0.05920612\right)+\left(\text{NFKBIZ}\times -0.0172617\right)+\left(\text{DLL}4\times 0.04204102\right)+\left(\text{RNASE}7\times 0.01673778\right)+\left(\text{IGKV}1-6\times 0.00041168\right)+\left(\text{IGLV}4-3\times 0.00150512\right)+\left(\text{IGLV}4-60\times 0.00203491\right)+\left(\text{SEMA}4\text{C}\times 0.01584736\right)+\left(\text{LTB}4\text{R}2\times -0.12208\right)+\left(\text{GREM}1\times -0.018175\right)+\left(\text{IL}33\times -0.0249882\right)+\left(\text{INHA}\times 0.00558738\right)+\left(\text{JAG}1\times 0.00402041\right)+\left(\text{NRTN}\times -0.0641765\right)+\left(\text{PDGFB}\times 0.01783257\right)+\left(\text{PNOC}\times 0.03268342\right)+\left(\text{FGFR}4\times 0.0362506\right)+\left(\text{GCGR}\times -0.1657741\right)+\left(\text{HNF}4\text{G}\times 0.06686767\right)+\left(\text{LGR}4\times 0.01716712\right)+\left(\text{SHC}3\times -0.1521834\right). \end{array}$$

**Figure 4:**
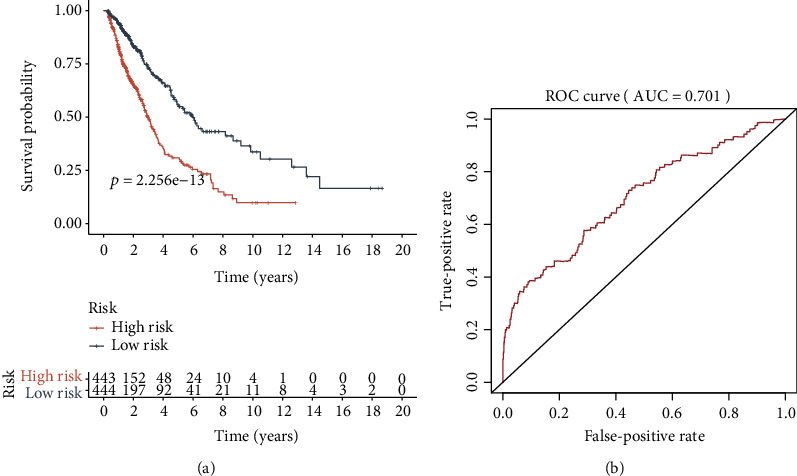
Prognostic analysis. (a) Kaplan-Meier curve analysis of overall survival (OS) in high-risk and low-risk groups; high-risk score patients demonstrated poor OS than those with a low-risk score. (b) Analysis via time-dependent receiver operating characteristic (ROC) curve for the prognostic model. AUC: area under the curve.

**Figure 5:**
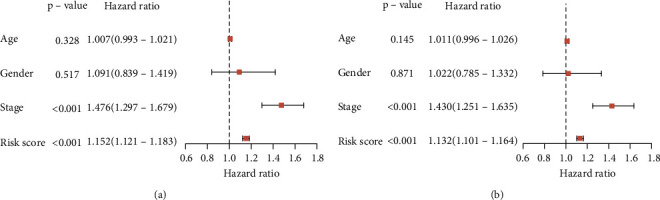
Univariate (a) and multivariate (b) Cox regression analyses of the clinical factors and risk score.

We stratified patients into low and high immune risk groups, using the median value of the gene set risk scores. Survival analysis depicted a great difference between the two groups (Figure 4(a)). ROC curve analysis showed a moderate potential for survival prediction (AUC: 0.701; Figure 4(b)). In multivariate analysis, together with additional clinical factors (gender, age, and pathological stage), the risk score remained as an independent prognostic signature (hazard ratio: 1.132; 95% confidence interval: 1.101–1.164; *P* < 0.001; Figure 5).

### 3.3. TF Regulatory Network

**Figure 6:**
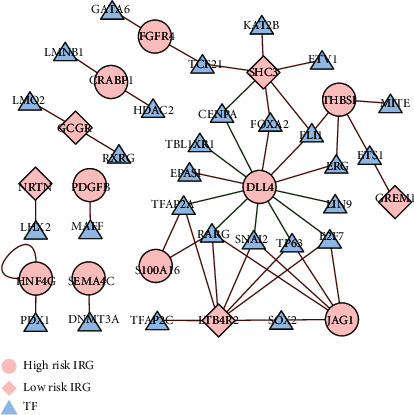
Regulatory network of transcription factors (TFs) and prognostic differentially expressed immune-related genes (IRGs); the diamond nodes and ellipse nodes denote IRGs with hazard ratio (HR) <1 and HR >1, respectively; the triangle nodes denote TFs that correlated with 26 IRGs (correlation coefficient >0.3 and *P* < 0.001). Red and green lines denote positive and negative regulation, respectively.

The two major protein networks were the protein-protein interaction network and the transcriptional regulation network. We screened 318 TFs derived from the Cistrome Cancer database and found differential expression between nontumor lung tissue and NSCLC samples in 86 TFs. We then constructed a gene regulatory network from the 86 TFs and 26 prognostic DEIRGs, based on their gene expression values (correlation coefficient threshold: ≥0.3; *P* < 0.001; Figure 6).

### 3.4. Interactions between the IRGPI with Tumor Immune Cell Infiltration and Clinicopathologic Parameters

**Figure 7:**
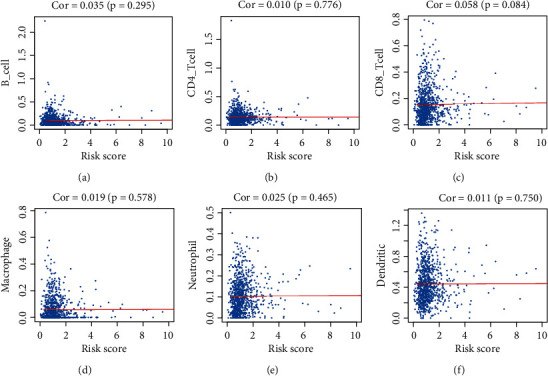
Analysis of the correlation between the risk score and infiltration degree of six immune cell types. (a) B cells. (b) CD4+ T cells. (c) CD8+ T cells. (d) Macrophages. (e) Neutrophils. (f) Dendritic cells.

To see if IRGPI reflected TIME status, we analyzed associations between risk scores and tumor-infiltrated immune cells (B cells, CD4^+^ T cells, CD8^+^ T cells, macrophages, neutrophils, and dendritic cells). Correlations were not apparent (Figure 7). We also investigated the expression differences of IRGPI between categorical Clinicopathologic parameters; the expression of S100A16, RNASE7, LTB4R2, and INHA were different at different pathological stages; PLAU, CRABP1, IGKV1-6, and INHA were differentially expressed at different T stages; RBP2, RNASE7, LTB4R2, and GCGR expression associated with distant metastasis; and IL33, PDGFB, PNOC, and SHC3 correlated with lymph node metastasis. The major result is the higher pathological stage was associated with a higher risk score (*P* = 0.013; Table 1).

## 4. Discussion

The immune system influences cancer initiation and progression and dysregulated immune contexture, and Immunoscore can affect oncologic outcomes [16, 17]. As the conventional TNM staging system provides limited prognostic information, combining immunological classifications with the American Joint Committee on Cancer/Union for International Cancer Control TNM staging system could greatly improve prognostic stratification. However, Immunoscore evaluates immune cell infiltration rather than the overall tumor immune status. The development of high-throughput RNA sequencing technology allows us to use immune-related gene expression to assess the status of immune cells and tumor cells, leading to more precise prognoses. Correlations between IRGs and lung adenocarcinoma prognosis have been explored by other studies [8, 9], but not for NSCLC. Therefore, developing a reliable IRG-based prognostic model of NSCLC and exploring the IRGs' respective clinical significances and molecular roles are critical.

In this study, we established a prognosis prediction model based on 26 IRGs. It showed moderate predictive ability (AUC: 0.701) and maintained its predictive ability independently from other clinical characteristics (gender, age, and pathological stage) and was strongly correlated with the clinicopathologic stage. Notably, our model indicates that fibroblast growth factor receptor 4 (FGFR4), a receptor tyrosine kinase, has potential as a therapeutic target in NSCLC, which has been also found in another study [18]. FGFR has been shown to play central functions in inflammation, embryogenesis, malignant tumor cell proliferation, and angiogenesis [19]. Studies have reported FGFR alterations in several solid malignancies, especially urothelial carcinoma, and it has recently become an object of targeted therapy. The FGFR inhibitors, erdafitinib, and rogaratinib have been approved for clinical practice [20–22]. Hepatocyte nuclear factor 4-gamma (HNF4G) belongs to the orphan nuclear receptor superfamily and has been shown to influence growth and invasiveness in bladder cancer [23]; its place in our high-risk score group suggests its function on NSCLC which deserves further exploration. Teng, Y. C. et al. found that retinoblastoma-binding protein-2(RBP2), a histone demethylase, promoted lung tumorigenesis and progression, and expression of integrin-*β*1, which is associated with lung cancer metastasis [24]; our current study supports this result. Increased expression of delta-like ligand 4 (DLL4), another IRG in our study, has been observed in many tumor types and may be related to worse outcomes [25–30]. DLL4-mediated Notch signaling signifies another key pathway for vascular development. Demcizumab, a humanized monoclonal antibody that inhibits DLL4 and interrupts Notch-mediated signaling, a phase IB trial has explored its feasibility combined with standard chemotherapy in metastatic nonsquamous NSCLC [31]. Subsequent randomized phase II clinical trials have shown some effect (NCT02259582). Dll4 blockade is a promising anti-angiogenesis therapy, particularly against resistant tumors [32].

Gene functional enrichment analyses suggest that the pathways implicated in DEIRGs are primarily associated with cytokine–cytokine receptor signaling pathways, which are crucial in angiogenesis, inflammatory processes, and chemotaxis [33]. A boosted inflammatory microenvironment is also a consistent feature in tumor progression and neoplastic processes [33, 34].

We constructed a TF-mediated network to discover molecular mechanisms of prognostic DEIRGs. TFs affect the prognosis mainly by regulating the expression of DLL4, THBS1, JAG1, SHC3, and LTB4R2. TCF-21 positively regulates FGFR4 according to our network, but conclusive evidence is not yet available. ERG interacts with Notch intracellular domain (NICD) and *β*-catenin and is required for Ang1-dependent *β*-catenin recruitment at the DLL4 locus [35]. The network also showed a strong correlation between LHX2 and NRTN, which implies an insight into changes in the NSCLC immune system at the molecular level.

High levels of tumor-infiltrating lymphocytes (TILs) are associated with better outcomes for patients with completely resected NSCLC [36]. Schalper et al. observed the prognostic value of CD8^+^ TILs in NSCLC [37]; other studies confirmed this result [38, 39]. However, Wakabayashi et al. suggest that CD4^+^ T cells in cancer stroma, rather than CD8^+^ T cells in cancer cell nests, are related to favorable prognosis in NSCLC patients [40]. These studies disagree on how tumor growth and prognosis are influenced by TILs. Few studies have explored the relationships between IRGPI and TILs in lung cancer, and results have not been consistent; although high neutrophil infiltration may predict worse outcomes [8, 11], and this correlation is not very strong [11]. We analyzed the relationships in our study to examine the immune microenvironment but found no significant correlation between them. Further studies are needed.

Taken together, our results show that IRGPI can both estimate the survival of NSCLC patients and indicate potential therapeutic targets for further study. Dysregulated IRGs may indicate variations in immunotherapy sensitivity, permitting individualized treatment strategies.

Inevitably, our research had several limitations. First, we used retrospective data to develop a signature from public databases. Second, clinical validation is needed to verify the signature's efficacy. Third, as we did not enroll patients who were treated with immune checkpoint inhibitors, we were unable to confirm any association between immunotherapy response and the signature.

Additional prospective studies are needed to validate our model's prognostic accuracy for survival and immunotherapy response in patients with NSCLC. Furthermore, our model's IRGs suggest novel molecular targets and prognostic biomarkers, which also warrant investigation and clinical translation.

## 5. Conclusion

In summary, we developed a robust model to predict the survival of patients with NSCLC, based on the expression of 26 IRGs, which can potentially augment TNM staging. Although our model needs further validation, it may provide novel predictive and therapeutic molecular targets in patients with NSCLC.

## Figures and Tables

**Table 1:** Relationships between model variables and clinical variables.

Variables	Age (≤65/>65)	Gender (female/male)	Stage (I-II/III-IV)	T (T1-2/T3-4)	M (M0/M1)	N (N0/N1-3)
	*t* (*P*)	*t* (*P*)	*t* (*P*)	*t* (*P*)	*t* (*P*)	*t* (*P*)
THBS1	0.452 (0.651)	1.918 (0.056)	-0.966 (0.336)	-1.187 (0.237)	1.936 (0.062)	-0.734 (0.463)
MMP12	*-3.32 (9.509e-04)*	-1.659 (0.098)	1.06 (0.290)	0.873 (0.384)	0.068 (0.946)	-0.061 (0.951)
S100A16	*-2.603 (0.009)*	*-2.947 (0.003)*	*-2.127 (0.035)*	-1.304 (0.194)	0.012 (0.990)	-1.59 (0.112)
PLAU	*-3.046 (0.002)*	0.166 (0.868)	-0.599 (0.550)	*-2.572 (0.011)*	1.441 (0.160)	0.482 (0.630)
CRABP1	1.256 (0.210)	0.745 (0.457)	-0.917 (0.361)	*2.124 (0.034)*	-1.167 (0.254)	1.839 (0.067)
RBP2	-1.044 (0.297)	1.913 (0.057)	1.37 (0.172)	0.623 (0.534)	*3.118 (0.002)*	0.629 (0.530)
NFKBIZ	0.437 (0.662)	1.577 (0.115)	0.868 (0.386)	0.343 (0.732)	1.588 (0.123)	0.637 (0.524)
DLL4	0.92 (0.358)	*3.875 (1.204e-04)*	-1.905 (0.058)	2.003 (0.047)	-0.947 (0.352)	-1.408 (0.160)
RNASE7	-1.695 (0.091)	*-2.162 (0.031)*	*2.742 (0.006)*	-1.474 (0.143)	*4.849 (2.731e-06)*	0.735 (0.463)
IGKV1-6	*-2.26 (0.024)*	0.472 (0.637)	1.804 (0.072)	*2.309 (0.021)*	1.637 (0.106)	1.809 (0.071)
IGLV4-3	-0.644 (0.520)	1.028 (0.304)	0.744 (0.457)	0.541 (0.589)	0.038 (0.970)	-0.128 (0.898)
IGLV4-60	-0.566 (0.572)	0.474 (0.636)	-0.166 (0.868)	0.942 (0.347)	0.82 (0.418)	0.255 (0.799)
SEMA4C	-0.533 (0.595)	1.161 (0.246)	-1.334 (0.184)	-0.698 (0.486)	0.771 (0.446)	-1.505 (0.133)
LTB4R2	0.274 (0.784)	*-3.348 (8.603e-04)*	*2.52 (0.012)*	-0.996 (0.321)	*2.552 (0.016)*	0.673 (0.501)
GREM1	-0.233 (0.816)	-0.646 (0.518)	0.494 (0.622)	-0.31 (0.757)	1.002 (0.325)	-1.157 (0.248)
IL33	-0.795 (0.427)	0.752 (0.452)	1.411 (0.159)	1.074 (0.285)	0.729 (0.471)	*2.886 (0.004)*
INHA	-0.383 (0.702)	0.611 (0.542)	*-2.036 (0.043)*	*2.434 (0.015)*	-1.987 (0.058)	-0.49 2(0.623)
JAG1	-1.526 (0.128)	*-4.898 (1.222e-06)*	-0.579 (0.564)	-1.352 (0.179)	1.281 (0.210)	-0.738 (0.461)
NRTN	0.492 (0.623)	-1.443 (0.150)	0.195 (0.846)	-0.309 (0.758)	1.639 (0.112)	-0.963 (0.336)
PDGFB	-1.432 (0.153)	-1.556 (0.120)	1.513 (0.131)	-1.331 (0.186)	0.848 (0.403)	*1.982 (0.048)*
PNOC	-0.263 (0.793)	1.816 (0.070)	1.8 (0.073)	0.548 (0.585)	1.215 (0.234)	*2.445 (0.015)*
FGFR4	0.23 (0.818)	-1.019 (0.309)	-0.692 (0.490)	0.228 (0.820)	0.254 (0.801)	-0.319 (0.750)
GCGR	0.352 (0.725)	-0.687 (0.492)	1.248 (0.212)	0.448 (0.655)	*4.649 (4.147e-06)*	0.457 (0.648)
HNF4G	0.968 (0.334)	1.35 (0.178)	-0.973 (0.332)	-0.208 (0.835)	-1.328 (0.195)	-0.332 (0.740)
LGR4	0.494 (0.621)	1.621 (0.106)	-1.264 (0.208)	0.227 (0.821)	-0.271 (0.789)	-0.361 (0.718)
SHC3	0.95 (0.343)	*4.899 (1.393e-06)*	-0.127 (0.899)	-0.456 (0.649)	-0.584 (0.565)	*2.464 (0.014)*
Risk score	-0.649 (0.517)	-0.367 (0.714)	*-2.525 (0.013)*	-1.215 (0.227)	-1.196 (0.243)	-1.531 (0.127)

Note: *t*: *t* value from Student's *t*-test; *P*: *P* value from Student's *t*-test; T: tumor; N: node; M: metastasis.

## Data Availability

We extracted RNA sequencing data and clinical information of NSCLC patients from the TCGA data portal (https://cancergenome.nih.gov/). In the ImmPort database (https://immport.niaid.nih.gov/), the IRGs list was retrieved, and TFs were retrieved from the Cistrome database (http://cistrome.org/). From TIMER online database (https://cistrome.shinyapps.io/timer/), we retrieved the information about the immune infiltrate of NCSLC patients. All data downloaded at 13 January 2020.

## Abbreviations

NSCLC: Nonsmall cell lung cancer
TCGA: The cancer genome atlas
IRGs: Immune-related genes
ImmPort: Immunology database and analysis portal
DEIRGs: Differentially expressed immune-related genes
OS: Overall survival
AUC: Area under the curve
TIMER: Tumor immune estimation resource
FDR: False discovery rate
KEGG: Kyoto Encyclopedia of Genes and Genomes
GO: Gene Ontology
IRGPI: Immune-related gene prognostic index
ROC: Receiver operating characteristic
TFs: Transcription factors
TIME: Tumor immune microenvironment
T: Tumor
N: Node
M: Metastasis
BP: Biological process
CC: Cellular components
MF: Molecular functions
HR: Hazard ratio
CI: Confidence interval
FGFR4: Fibroblast growth factor receptor 4
HNF4G: Hepatocyte nuclear factor 4 gamma
RBP2: Retinoblastoma-binding protein-2
Dll4: Delta-like ligand 4
TILs: Tumor-infiltrating lymphocytes
FC: Fold change.

## Supplementary Materials

Supplementary Materials Supplementary Table 1: The DEIRGs between adjacent nontumor and tumor tissues.
