# Supplementary material for: Prognostic Index for Nonsmall Cell Lung Cancer Based on Immune-Related Genes Expression
Source: Biomed Res Int. 2022 Sep 19;2022:4779811. doi: 10.1155/2022/4779811 (PMC9526605; doi:10.1155/2022/4779811)
Supplement: Supplementary Materials — Supplementary Table 1: The DEIRGs between adjacent nontumor and tumor tissues. [file 4779811.f1.docx]

Supplementary Table 1. The DEIRGs between adjacent non-tumor and tumor tissues

| Gene symbol | Mean | | logFC | *P*-value | FDR |
| --- | --- | --- | --- | --- | --- |
|  | Control | Tumor |  |  |  |
| CD1A | 1.23970204 | 3.50400186 | 1.49901013 | 0.00071724 | 0.00087486 |
| CD4 | 43.6174796 | 21.6929417 | -1.0076807 | 2.80E-37 | 1.69E-36 |
| CD74 | 1919.38924 | 948.0484 | -1.0176147 | 3.75E-34 | 1.90E-33 |
| CTSS | 115.070657 | 51.7615531 | -1.1525672 | 1.13E-40 | 8.40E-40 |
| FCER1G | 124.54054 | 51.2128873 | -1.2820366 | 5.35E-45 | 5.30E-44 |
| HLA-DOA | 33.7200343 | 14.232948 | -1.2443715 | 7.61E-40 | 5.36E-39 |
| HLA-DPA1 | 173.211267 | 74.1678971 | -1.223666 | 5.05E-40 | 3.60E-39 |
| HLA-DPB1 | 271.244064 | 122.633914 | -1.1452336 | 5.31E-39 | 3.54E-38 |
| HLA-DQA2 | 73.5799972 | 33.2596824 | -1.1455392 | 5.86E-12 | 1.06E-11 |
| HLA-DQB1 | 106.232096 | 52.7365249 | -1.0103453 | 9.02E-25 | 2.90E-24 |
| HLA-DRA | 2267.83273 | 967.408966 | -1.2291164 | 2.58E-39 | 1.76E-38 |
| HLA-DRB1 | 1193.20644 | 521.175342 | -1.1950029 | 5.25E-38 | 3.29E-37 |
| HLA-DRB5 | 505.683848 | 194.842835 | -1.3759248 | 4.02E-33 | 1.94E-32 |
| HLA-E | 709.080694 | 273.608559 | -1.3738365 | 1.26E-57 | 4.74E-56 |
| HSPA1B | 23.2924967 | 48.6533012 | 1.06267241 | 9.18E-26 | 3.09E-25 |
| HSPA6 | 3.1221802 | 7.42453067 | 1.24974603 | 2.11E-07 | 3.08E-07 |
| ICAM1 | 180.064427 | 66.6030527 | -1.434853 | 4.05E-29 | 1.60E-28 |
| KLRD1 | 0.64904102 | 0.3029733 | -1.099119 | 2.56E-30 | 1.07E-29 |
| PSMC4 | 22.8601853 | 51.9423799 | 1.18407502 | 1.86E-54 | 4.33E-53 |
| PSMD2 | 28.7895852 | 76.9342833 | 1.41807963 | 7.43E-44 | 6.75E-43 |
| PSMD11 | 9.92535722 | 22.7315439 | 1.19550474 | 3.42E-52 | 6.23E-51 |
| THBS1 | 81.358667 | 39.8439882 | -1.029934 | 3.07E-14 | 6.18E-14 |
| PSMD14 | 5.39652355 | 12.1553295 | 1.17148677 | 2.30E-51 | 3.87E-50 |
| ULBP3 | 0.93750434 | 2.0312505 | 1.1154709 | 1.18E-11 | 2.10E-11 |
| ULBP2 | 1.44765173 | 5.6348494 | 1.96066248 | 1.63E-06 | 2.29E-06 |
| RAET1L | 0.01907727 | 4.01473806 | 7.7173075 | 1.08E-27 | 3.95E-27 |
| PDIA2 | 0.08002499 | 1.83622235 | 4.52014641 | 1.37E-25 | 4.58E-25 |
| HAMP | 0.27602972 | 0.56662476 | 1.03757004 | 8.37E-15 | 1.72E-14 |
| PI3 | 4.71041127 | 237.507406 | 5.65597566 | 1.07E-06 | 1.51E-06 |
| CAMP | 4.06915643 | 0.70003665 | -2.5392274 | 2.12E-53 | 4.40E-52 |
| PPBP | 10.082311 | 1.69714761 | -2.5706424 | 1.75E-48 | 2.26E-47 |
| CXCL14 | 4.74226956 | 89.7439019 | 4.24216434 | 1.93E-30 | 8.12E-30 |
| CXCL16 | 96.6190821 | 40.9327159 | -1.2390538 | 3.44E-51 | 5.68E-50 |
| SLPI | 1324.72005 | 570.33311 | -1.2158108 | 2.27E-39 | 1.55E-38 |
| CXCL10 | 16.8922266 | 41.9325491 | 1.31171103 | 1.95E-05 | 2.59E-05 |
| CXCL9 | 14.3264539 | 36.8904029 | 1.36456399 | 6.28E-05 | 8.11E-05 |
| CXCL6 | 1.45430191 | 7.42919254 | 2.35287862 | 7.05E-05 | 9.08E-05 |
| CXCL12 | 15.7601508 | 6.14846487 | -1.3579832 | 1.38E-41 | 1.08E-40 |
| CXCL13 | 7.81021686 | 44.0611246 | 2.49607181 | 9.67E-32 | 4.33E-31 |
| CXCL2 | 137.004199 | 19.137977 | -2.8397099 | 2.86E-38 | 1.82E-37 |
| PF4 | 2.63576973 | 0.41881725 | -2.6538316 | 6.15E-46 | 6.57E-45 |
| CXCL3 | 11.1633705 | 2.74102984 | -2.0259827 | 2.65E-28 | 1.00E-27 |
| LCN2 | 24.1834057 | 102.640949 | 2.08551708 | 1.16E-09 | 1.89E-09 |
| COLEC10 | 1.16334825 | 0.42995802 | -1.4360153 | 1.54E-41 | 1.21E-40 |
| MMP12 | 1.08402095 | 36.7910955 | 5.08489209 | 1.35E-50 | 2.10E-49 |
| SFTPD | 667.877072 | 106.161315 | -2.6533244 | 3.39E-57 | 1.18E-55 |
| PTGDS | 96.2061054 | 25.0849505 | -1.9393064 | 1.86E-51 | 3.16E-50 |
| S100B | 2.84493027 | 5.77303694 | 1.02093716 | 0.00360867 | 0.00423247 |
| S100P | 6.4427469 | 117.631071 | 4.19044945 | 6.42E-12 | 1.16E-11 |
| S100A7 | 0.30012672 | 99.9261482 | 8.37914668 | 2.63E-15 | 5.53E-15 |
| DEFB126 | 0.00453398 | 0.28458631 | 5.9719449 | 7.75E-19 | 1.90E-18 |
| PGLYRP3 | 0.01882064 | 3.74810649 | 7.63770207 | 4.04E-42 | 3.29E-41 |
| S100A2 | 3.37578816 | 235.641751 | 6.12522699 | 1.35E-38 | 8.79E-38 |
| LCN12 | 0.14389516 | 0.65342683 | 2.1830076 | 1.18E-18 | 2.88E-18 |
| PGLYRP4 | 0.05836551 | 1.95953204 | 5.06924929 | 1.20E-39 | 8.31E-39 |
| S100A11 | 549.621178 | 1114.49447 | 1.01987996 | 2.79E-30 | 1.17E-29 |
| S100A5 | 0.3152444 | 1.17576842 | 1.89906128 | 5.02E-11 | 8.69E-11 |
| S100A3 | 9.84448403 | 3.00579898 | -1.7115671 | 4.78E-33 | 2.30E-32 |
| S100A7A | 0.0073953 | 1.64369754 | 7.79611962 | 9.87E-12 | 1.77E-11 |
| COLEC12 | 18.8413294 | 4.82516078 | -1.9652518 | 3.81E-57 | 1.32E-55 |
| S100A14 | 76.4695906 | 166.660007 | 1.12394989 | 3.03E-07 | 4.40E-07 |
| S100A16 | 52.720325 | 168.110845 | 1.67298163 | 1.04E-33 | 5.16E-33 |
| ZC3HAV1L | 1.10798467 | 4.22345341 | 1.93048522 | 1.16E-48 | 1.52E-47 |
| WFDC2 | 77.5685329 | 184.160814 | 1.2474227 | 0.00351792 | 0.0041289 |
| IL6 | 38.3671996 | 5.93011589 | -2.6937413 | 4.27E-22 | 1.22E-21 |
| UMODL1 | 0.0491873 | 0.66141653 | 3.74920131 | 2.52E-11 | 4.42E-11 |
| MMP9 | 9.46668971 | 47.9695508 | 2.34118699 | 6.79E-29 | 2.65E-28 |
| TLR4 | 11.1108827 | 3.55979456 | -1.6421076 | 4.66E-53 | 9.37E-52 |
| A2M | 679.818048 | 133.36567 | -2.3497613 | 5.53E-60 | 3.58E-58 |
| PLAU | 11.0592613 | 77.0970344 | 2.80142034 | 2.74E-42 | 2.26E-41 |
| PAEP | 0.2163803 | 33.27992 | 7.264939 | 1.18E-16 | 2.63E-16 |
| OBP2A | 0.0504066 | 0.64365767 | 3.67460911 | 0.00090529 | 0.00109805 |
| SFTPA2 | 7341.69773 | 864.484791 | -3.0862012 | 4.20E-59 | 2.15E-57 |
| LBP | 0.30221378 | 2.94225264 | 3.28327976 | 1.45E-05 | 1.94E-05 |
| RBP4 | 18.4131025 | 4.12123775 | -2.1595831 | 4.29E-51 | 7.04E-50 |
| SFTPA1 | 6877.51477 | 709.376127 | -3.2772646 | 4.64E-61 | 4.15E-59 |
| NOX4 | 0.37112217 | 1.02183952 | 1.46120254 | 9.48E-22 | 2.66E-21 |
| FABP7 | 0.02472224 | 3.83414652 | 7.27695212 | 1.11E-15 | 2.38E-15 |
| FABP4 | 98.7258381 | 7.04164149 | -3.809444 | 1.42E-61 | 1.62E-59 |
| CRABP2 | 4.25555937 | 142.776486 | 5.06826582 | 6.78E-54 | 1.48E-52 |
| CRABP1 | 0.11910766 | 4.66110974 | 5.29033537 | 1.09E-24 | 3.48E-24 |
| DUOX1 | 26.8020331 | 6.09637741 | -2.1363183 | 3.80E-57 | 1.31E-55 |
| RBP2 | 3.70823739 | 0.45575396 | -3.0244065 | 4.75E-56 | 1.38E-54 |
| CETP | 2.18678096 | 0.81058199 | -1.4317787 | 1.99E-38 | 1.28E-37 |
| C8G | 0.32054869 | 0.95939185 | 1.58157665 | 1.90E-12 | 3.52E-12 |
| PI15 | 0.34026148 | 1.73059065 | 2.34654875 | 9.09E-29 | 3.53E-28 |
| NOX1 | 0.28203825 | 0.68921309 | 1.28905928 | 1.86E-19 | 4.70E-19 |
| CTSG | 3.70710322 | 0.93677803 | -1.9845131 | 9.52E-31 | 4.07E-30 |
| CYBB | 61.3275937 | 22.1842182 | -1.4670026 | 1.26E-40 | 9.36E-40 |
| SOCS3 | 172.909191 | 59.3596873 | -1.5424592 | 7.44E-20 | 1.92E-19 |
| TLR7 | 2.40673435 | 1.13341312 | -1.0864031 | 6.38E-31 | 2.74E-30 |
| IKBKE | 2.47216467 | 6.8643797 | 1.47335451 | 1.14E-47 | 1.38E-46 |
| ISG15 | 40.1567634 | 92.1150988 | 1.19779466 | 9.80E-11 | 1.67E-10 |
| TFR2 | 0.09217997 | 0.88671017 | 3.26593743 | 1.28E-58 | 5.76E-57 |
| MUC4 | 1.28878254 | 6.35193343 | 2.30118694 | 2.87E-10 | 4.79E-10 |
| ELN | 27.8699576 | 13.5309477 | -1.0424479 | 1.81E-24 | 5.76E-24 |
| LYZ | 602.43738 | 248.88687 | -1.2753212 | 1.07E-37 | 6.58E-37 |
| CST4 | 0.00654504 | 1.34480749 | 7.68278213 | 4.30E-40 | 3.09E-39 |
| ITGAV | 13.5236568 | 28.6383514 | 1.08246313 | 5.11E-28 | 1.90E-27 |
| TLR8 | 5.59904798 | 1.58274115 | -1.8227562 | 2.96E-48 | 3.76E-47 |
| CACYBP | 8.80721288 | 19.9025228 | 1.17619387 | 1.21E-50 | 1.89E-49 |
| BPHL | 1.4136643 | 3.30880339 | 1.22687 | 2.91E-45 | 2.95E-44 |
| WNT5A | 3.60949872 | 10.8081302 | 1.58224656 | 1.47E-06 | 2.07E-06 |
| AHNAK | 123.813213 | 41.7730362 | -1.5675214 | 2.45E-48 | 3.13E-47 |
| FGF2 | 2.54741975 | 0.88004866 | -1.5333815 | 4.30E-44 | 3.98E-43 |
| F2RL1 | 3.98892996 | 11.8038844 | 1.565188 | 3.82E-26 | 1.30E-25 |
| MSR1 | 35.9428511 | 6.78999426 | -2.4042226 | 3.04E-58 | 1.27E-56 |
| NFKBIZ | 20.419851 | 9.2540033 | -1.1418228 | 3.25E-15 | 6.83E-15 |
| PDF | 0.67130244 | 1.66907512 | 1.3140141 | 1.88E-45 | 1.94E-44 |
| DLL4 | 9.54913253 | 3.99932684 | -1.2556125 | 4.75E-35 | 2.52E-34 |
| SLC11A1 | 13.8841684 | 3.56531372 | -1.9613398 | 2.76E-51 | 4.60E-50 |
| DMBT1 | 56.0226411 | 17.8620788 | -1.64911 | 2.94E-35 | 1.58E-34 |
| DES | 40.619589 | 3.79754644 | -3.4190361 | 1.65E-60 | 1.23E-58 |
| MARCO | 203.699186 | 21.7482467 | -3.2274692 | 1.07E-60 | 8.37E-59 |
| TNFSF11 | 0.09052378 | 0.81735329 | 3.17459105 | 1.44E-41 | 1.13E-40 |
| JUND | 175.066003 | 65.0517461 | -1.4282393 | 6.02E-43 | 5.19E-42 |
| CLDN4 | 38.1653386 | 78.7395392 | 1.04482528 | 6.85E-17 | 1.55E-16 |
| LTB4R | 1.7484118 | 5.89504324 | 1.75345737 | 1.51E-15 | 3.22E-15 |
| IL7R | 36.5553366 | 9.67618771 | -1.9175714 | 3.63E-45 | 3.64E-44 |
| PTX3 | 7.41522101 | 1.69795041 | -2.1266954 | 1.32E-24 | 4.21E-24 |
| SYTL1 | 4.6737442 | 10.5925141 | 1.18039436 | 1.21E-24 | 3.88E-24 |
| CD40LG | 1.77681679 | 0.83596132 | -1.0877868 | 1.92E-20 | 5.09E-20 |
| MASP1 | 1.31315236 | 0.24783043 | -2.405609 | 4.59E-57 | 1.56E-55 |
| PROC | 0.12199208 | 1.4674576 | 3.58845947 | 4.68E-42 | 3.79E-41 |
| NDRG1 | 39.6132947 | 120.609332 | 1.60628493 | 5.81E-15 | 1.20E-14 |
| HMOX1 | 77.0963445 | 31.2859828 | -1.301146 | 4.33E-17 | 9.87E-17 |
| RNASE7 | 0.06232007 | 2.08675198 | 5.06541855 | 1.04E-06 | 1.47E-06 |
| HGF | 3.07741578 | 1.34020076 | -1.1992702 | 5.59E-41 | 4.24E-40 |
| HDAC1 | 19.5382233 | 42.7946357 | 1.13113068 | 2.42E-55 | 6.30E-54 |
| ARRB1 | 21.1311907 | 6.54834188 | -1.6901725 | 5.88E-54 | 1.30E-52 |
| PCSK1 | 0.09256931 | 6.14932366 | 6.05375004 | 1.50E-26 | 5.21E-26 |
| ARG2 | 1.29043444 | 3.36779276 | 1.38394652 | 7.28E-21 | 1.97E-20 |
| AQP9 | 12.830127 | 3.93047867 | -1.7067585 | 1.87E-41 | 1.46E-40 |
| BIRC5 | 0.86193264 | 18.3471999 | 4.41184096 | 5.34E-61 | 4.60E-59 |
| VIM | 334.228274 | 133.137227 | -1.3279198 | 1.55E-54 | 3.64E-53 |
| PRDX1 | 154.441374 | 312.479816 | 1.01670371 | 1.22E-25 | 4.08E-25 |
| AGER | 1013.06919 | 27.4112918 | -5.2078186 | 5.18E-65 | 1.17E-61 |
| TNFSF4 | 0.58009829 | 1.41629107 | 1.28774853 | 4.22E-19 | 1.05E-18 |
| CCL14 | 2.2775642 | 0.32951775 | -2.7890636 | 1.77E-51 | 3.00E-50 |
| CCL19 | 16.2921807 | 35.5659822 | 1.12631828 | 1.74E-05 | 2.32E-05 |
| CCL18 | 168.996224 | 76.6407944 | -1.1408066 | 2.56E-14 | 5.17E-14 |
| CCL26 | 0.29194429 | 2.29686927 | 2.97590372 | 4.40E-23 | 1.32E-22 |
| CCR3 | 0.10613995 | 0.24207457 | 1.18948372 | 5.46E-05 | 7.07E-05 |
| CCR8 | 0.23236146 | 0.63787183 | 1.45689577 | 5.12E-19 | 1.27E-18 |
| CCL2 | 83.9746198 | 23.2634109 | -1.8518907 | 1.37E-18 | 3.32E-18 |
| CCL21 | 98.2735344 | 48.554929 | -1.0171852 | 4.52E-19 | 1.12E-18 |
| CCL7 | 0.3457868 | 1.15172264 | 1.73583862 | 2.07E-15 | 4.38E-15 |
| CCL23 | 8.58492616 | 1.17662185 | -2.867155 | 3.19E-58 | 1.32E-56 |
| CCL25 | 0.03745861 | 0.30359048 | 3.01875744 | 1.51E-22 | 4.41E-22 |
| CCR1 | 13.7977175 | 6.42654102 | -1.1023153 | 7.84E-32 | 3.53E-31 |
| CCL24 | 12.1655717 | 2.35727406 | -2.3676127 | 2.50E-22 | 7.21E-22 |
| CDH1 | 34.2829088 | 77.8135961 | 1.18253273 | 1.49E-38 | 9.68E-38 |
| PPARG | 12.4038843 | 4.02221972 | -1.6247282 | 7.03E-50 | 1.03E-48 |
| FGR | 25.3014011 | 5.98606365 | -2.0795378 | 1.74E-59 | 9.73E-58 |
| MIF | 16.3062553 | 58.0004289 | 1.83063806 | 6.26E-49 | 8.43E-48 |
| HCK | 28.777234 | 13.7348857 | -1.067083 | 4.88E-39 | 3.27E-38 |
| OLR1 | 62.5031094 | 9.61942419 | -2.6999055 | 7.89E-59 | 3.76E-57 |
| CD79A | 10.6355825 | 21.9371303 | 1.04447576 | 3.91E-13 | 7.47E-13 |
| BTK | 7.81707109 | 2.72944329 | -1.5180214 | 2.64E-51 | 4.42E-50 |
| RAC3 | 1.77871854 | 11.6448821 | 2.71078589 | 3.39E-52 | 6.18E-51 |
| FOS | 320.716299 | 87.352546 | -1.876376 | 6.21E-33 | 2.96E-32 |
| CARD11 | 2.27792689 | 7.37539194 | 1.69499827 | 3.13E-18 | 7.49E-18 |
| NFKBIA | 198.867457 | 93.6518249 | -1.0864282 | 8.06E-30 | 3.30E-29 |
| PIK3R5 | 4.71762681 | 1.86744178 | -1.336998 | 7.16E-43 | 6.13E-42 |
| PIK3R2 | 0.12023563 | 0.2866012 | 1.2531802 | 2.47E-40 | 1.80E-39 |
| LILRB3 | 3.16741428 | 1.14665481 | -1.4658744 | 1.27E-48 | 1.66E-47 |
| IGHA1 | 1330.37548 | 3125.0991 | 1.23206846 | 1.71E-07 | 2.51E-07 |
| IGHA2 | 118.87544 | 326.320187 | 1.45683755 | 5.19E-09 | 8.16E-09 |
| IGHD2-2 | 0.51088533 | 2.07101575 | 2.01926712 | 2.84E-09 | 4.52E-09 |
| IGHD3-22 | 0.81574327 | 2.69275599 | 1.72289642 | 6.05E-10 | 9.93E-10 |
| IGHD3-3 | 1.15685173 | 5.22868353 | 2.17624379 | 2.03E-11 | 3.57E-11 |
| IGHD3-9 | 1.10050427 | 3.63740195 | 1.72474362 | 1.38E-07 | 2.03E-07 |
| IGHD6-25 | 0.68946548 | 2.10554162 | 1.61064118 | 3.72E-06 | 5.14E-06 |
| IGHG1 | 309.765433 | 1378.57871 | 2.15393357 | 2.26E-22 | 6.55E-22 |
| IGHG2 | 171.122202 | 828.083339 | 2.27474901 | 8.04E-24 | 2.49E-23 |
| IGHG3 | 105.112581 | 474.005528 | 2.17296853 | 5.13E-19 | 1.27E-18 |
| IGHG4 | 65.261075 | 520.638749 | 2.99598803 | 2.64E-31 | 1.16E-30 |
| IGHJ1 | 2.18381164 | 9.71353129 | 2.15314745 | 5.30E-12 | 9.61E-12 |
| IGHJ2 | 7.46226817 | 30.4137031 | 2.02703538 | 5.64E-14 | 1.12E-13 |
| IGHJ3 | 17.453485 | 59.1853069 | 1.76172393 | 5.44E-15 | 1.13E-14 |
| IGHM | 165.066209 | 432.228708 | 1.38875009 | 5.92E-08 | 8.89E-08 |
| IGHV1-18 | 60.4835763 | 280.12476 | 2.21145416 | 9.79E-16 | 2.11E-15 |
| IGHV1-2 | 34.0815906 | 134.656578 | 1.98222013 | 1.24E-11 | 2.20E-11 |
| IGHV1-24 | 31.1226074 | 125.027004 | 2.00620489 | 4.61E-17 | 1.05E-16 |
| IGHV1-3 | 1.8744077 | 9.1686901 | 2.29028085 | 6.41E-10 | 1.05E-09 |
| IGHV1-45 | 3.55382254 | 15.2772666 | 2.1039429 | 0.00026853 | 0.00033552 |
| IGHV1-46 | 20.8093226 | 84.3354666 | 2.01890947 | 3.91E-15 | 8.17E-15 |
| IGHV1-58 | 5.49195925 | 20.6409598 | 1.91011723 | 7.10E-11 | 1.22E-10 |
| IGHV1-69 | 12.3412312 | 48.7125757 | 1.98080793 | 3.60E-12 | 6.58E-12 |
| IGHV2-26 | 11.9475726 | 45.4566753 | 1.92777464 | 3.90E-13 | 7.45E-13 |
| IGHV2-5 | 7.9121513 | 25.0574915 | 1.66310008 | 1.40E-10 | 2.36E-10 |
| IGHV2-70 | 13.2418588 | 40.9034406 | 1.62711655 | 3.51E-10 | 5.83E-10 |
| IGHV3-11 | 39.8740889 | 114.235462 | 1.51848711 | 1.75E-12 | 3.23E-12 |
| IGHV3-13 | 8.52425149 | 27.0633367 | 1.66669466 | 5.92E-07 | 8.48E-07 |
| IGHV3-15 | 45.2691581 | 161.287485 | 1.83303412 | 1.66E-12 | 3.09E-12 |
| IGHV3-16 | 0.09688728 | 0.33650979 | 1.79626927 | 1.13E-06 | 1.59E-06 |
| IGHV3-20 | 3.28839055 | 10.2154931 | 1.63530529 | 1.10E-09 | 1.79E-09 |
| IGHV3-21 | 38.057404 | 149.05318 | 1.9695781 | 1.56E-14 | 3.18E-14 |
| IGHV3-23 | 84.9207816 | 322.025399 | 1.92298493 | 3.65E-15 | 7.64E-15 |
| IGHV3-30 | 45.0896483 | 147.111471 | 1.70604159 | 1.55E-14 | 3.16E-14 |
| IGHV3-33 | 22.9357677 | 93.3567364 | 2.02515493 | 5.68E-16 | 1.23E-15 |
| IGHV3-35 | 1.00388106 | 3.1260677 | 1.63876068 | 1.07E-11 | 1.91E-11 |
| IGHV3-38 | 0.66016589 | 1.97631413 | 1.58191178 | 7.66E-12 | 1.38E-11 |
| IGHV3-43 | 6.15766157 | 22.8148022 | 1.88951566 | 1.70E-14 | 3.45E-14 |
| IGHV3-48 | 10.4277944 | 39.2056655 | 1.9106281 | 1.07E-12 | 2.01E-12 |
| IGHV3-49 | 20.746143 | 81.9706851 | 1.98226491 | 8.98E-11 | 1.53E-10 |
| IGHV3-53 | 7.38439013 | 30.978139 | 2.0686998 | 3.08E-13 | 5.90E-13 |
| IGHV3-64 | 2.23514561 | 9.97469652 | 2.15790413 | 8.94E-10 | 1.46E-09 |
| IGHV3-66 | 4.2361674 | 17.167954 | 2.01888661 | 1.89E-12 | 3.49E-12 |
| IGHV3-7 | 2.66864727 | 8.4382753 | 1.66083952 | 1.99E-09 | 3.19E-09 |
| IGHV3-72 | 7.06643287 | 24.3252062 | 1.783398 | 1.08E-06 | 1.53E-06 |
| IGHV3-73 | 11.1730149 | 61.8880106 | 2.46964141 | 1.90E-12 | 3.52E-12 |
| IGHV3-74 | 27.1789545 | 74.9679599 | 1.46378418 | 3.55E-10 | 5.90E-10 |
| IGHV4-28 | 5.98575803 | 18.6051659 | 1.63609739 | 1.18E-13 | 2.32E-13 |
| IGHV4-31 | 18.7134132 | 61.6401208 | 1.71979697 | 8.18E-12 | 1.47E-11 |
| IGHV4-34 | 33.4616552 | 135.77805 | 2.02066955 | 1.22E-17 | 2.85E-17 |
| IGHV4-39 | 71.0439182 | 236.039639 | 1.7322461 | 1.81E-13 | 3.51E-13 |
| IGHV4-4 | 3.31606069 | 11.668906 | 1.81512699 | 3.63E-10 | 6.02E-10 |
| IGHV4-59 | 41.4708047 | 145.616666 | 1.81200753 | 1.02E-12 | 1.92E-12 |
| IGHV4-61 | 5.4374568 | 16.9931536 | 1.64394968 | 1.25E-10 | 2.11E-10 |
| IGHV5-51 | 73.3969251 | 324.05425 | 2.14244383 | 2.07E-15 | 4.37E-15 |
| IGHV6-1 | 2.35251399 | 9.10761062 | 1.95286931 | 4.95E-12 | 8.98E-12 |
| IGHV7-81 | 0.50846657 | 2.24939268 | 2.1453107 | 7.59E-19 | 1.86E-18 |
| IGKC | 481.022773 | 1960.736 | 2.0272182 | 1.41E-19 | 3.58E-19 |
| IGKJ5 | 3.50407112 | 16.4632658 | 2.23214658 | 1.10E-20 | 2.95E-20 |
| IGKV1-12 | 3.44388236 | 7.92690427 | 1.20272169 | 2.54E-07 | 3.70E-07 |
| IGKV1-13 | 0.36939188 | 1.77135712 | 2.26163103 | 7.19E-12 | 1.30E-11 |
| IGKV1-16 | 21.5827479 | 103.211754 | 2.25765681 | 1.58E-11 | 2.80E-11 |
| IGKV1-17 | 21.634471 | 86.6786602 | 2.00234501 | 1.14E-12 | 2.14E-12 |
| IGKV1-27 | 30.1591676 | 73.830115 | 1.29161279 | 3.20E-09 | 5.08E-09 |
| IGKV1-33 | 0.89138852 | 4.23835489 | 2.24937812 | 4.97E-16 | 1.08E-15 |
| IGKV1-37 | 0.04204552 | 0.27519303 | 2.71042008 | 2.26E-12 | 4.17E-12 |
| IGKV1-39 | 2.10619313 | 9.11370382 | 2.11339976 | 1.71E-13 | 3.32E-13 |
| IGKV1-5 | 109.313564 | 431.68339 | 1.98150115 | 8.64E-15 | 1.78E-14 |
| IGKV1-6 | 18.0789483 | 76.6131336 | 2.08328098 | 1.44E-12 | 2.68E-12 |
| IGKV1-8 | 4.33325523 | 12.4253872 | 1.5197677 | 6.45E-14 | 1.28E-13 |
| IGKV1-9 | 34.9609431 | 124.192039 | 1.82875669 | 1.74E-13 | 3.37E-13 |
| IGKV1D-12 | 1.64682819 | 3.79522976 | 1.20449718 | 6.75E-10 | 1.11E-09 |
| IGKV1D-13 | 3.63835218 | 9.79234405 | 1.42836905 | 1.90E-05 | 2.53E-05 |
| IGKV1D-16 | 3.17136009 | 16.8556181 | 2.41005594 | 8.93E-08 | 1.33E-07 |
| IGKV1D-17 | 0.90952871 | 4.51269695 | 2.31079881 | 4.89E-07 | 7.04E-07 |
| IGKV1D-33 | 0.24900904 | 1.93929824 | 2.96126464 | 3.54E-12 | 6.48E-12 |
| IGKV1D-39 | 1.49518326 | 5.35208516 | 1.83977875 | 4.50E-12 | 8.19E-12 |
| IGKV1D-42 | 0.98538134 | 2.76614596 | 1.48912323 | 2.54E-08 | 3.87E-08 |
| IGKV1D-43 | 0.8493207 | 2.3937672 | 1.49490153 | 2.55E-07 | 3.72E-07 |
| IGKV1D-8 | 2.18313407 | 7.3757499 | 1.75638901 | 4.04E-12 | 7.36E-12 |
| IGKV2-24 | 14.8945542 | 59.5647224 | 1.99967319 | 5.93E-08 | 8.90E-08 |
| IGKV2-28 | 0.93808072 | 3.80291448 | 2.01932152 | 1.52E-11 | 2.70E-11 |
| IGKV2-30 | 3.38178537 | 12.0906673 | 1.83803686 | 4.13E-07 | 5.95E-07 |
| IGKV2-40 | 0.07522513 | 0.36307129 | 2.27096633 | 1.25E-08 | 1.93E-08 |
| IGKV2D-24 | 1.23473879 | 4.29791001 | 1.79942941 | 2.62E-06 | 3.64E-06 |
| IGKV2D-28 | 0.2407801 | 1.78916917 | 2.89350176 | 3.24E-10 | 5.38E-10 |
| IGKV2D-29 | 14.5029834 | 33.1434774 | 1.19237526 | 8.75E-09 | 1.36E-08 |
| IGKV2D-30 | 0.51537466 | 2.09466206 | 2.02302401 | 7.77E-06 | 1.05E-05 |
| IGKV2D-40 | 5.11420801 | 17.3585074 | 1.76306015 | 1.01E-10 | 1.72E-10 |
| IGKV3-11 | 96.4022228 | 378.939075 | 1.9748276 | 1.36E-17 | 3.16E-17 |
| IGKV3-15 | 41.159598 | 170.218478 | 2.04808686 | 3.25E-20 | 8.52E-20 |
| IGKV3-20 | 196.421359 | 653.017432 | 1.73316969 | 1.23E-15 | 2.64E-15 |
| IGKV3-7 | 2.44872745 | 9.24142976 | 1.91608386 | 2.69E-15 | 5.65E-15 |
| IGKV3D-11 | 2.43360314 | 9.23115812 | 1.92341774 | 3.82E-14 | 7.64E-14 |
| IGKV3D-15 | 2.63288633 | 12.3202539 | 2.22631484 | 1.81E-16 | 4.02E-16 |
| IGKV3D-20 | 7.46395672 | 29.9457215 | 2.00433737 | 3.14E-11 | 5.49E-11 |
| IGKV3D-7 | 0.26028293 | 0.9851509 | 1.92026406 | 1.22E-11 | 2.18E-11 |
| IGKV4-1 | 134.933774 | 567.848027 | 2.07325337 | 1.82E-17 | 4.23E-17 |
| IGKV5-2 | 3.18398038 | 12.6137739 | 1.98609663 | 5.42E-08 | 8.15E-08 |
| IGKV6-21 | 5.30007818 | 20.5391883 | 1.95429363 | 2.00E-09 | 3.21E-09 |
| IGKV6D-21 | 2.74667205 | 9.09170276 | 1.72686585 | 5.15E-05 | 6.67E-05 |
| IGKV6D-41 | 0.09401204 | 0.58373129 | 2.63438692 | 3.02E-07 | 4.38E-07 |
| IGLC2 | 276.701314 | 921.826064 | 1.73616506 | 3.50E-16 | 7.65E-16 |
| IGLC3 | 169.417342 | 608.604818 | 1.84492419 | 1.69E-15 | 3.59E-15 |
| IGLC6 | 1.00845336 | 3.17323115 | 1.65380826 | 1.31E-13 | 2.57E-13 |
| IGLC7 | 7.1223699 | 28.6857455 | 2.00990474 | 8.28E-08 | 1.23E-07 |
| IGLJ1 | 0.22264463 | 0.94365542 | 2.08351731 | 5.14E-14 | 1.02E-13 |
| IGLJ2 | 0.6047488 | 2.16863164 | 1.84237713 | 7.80E-17 | 1.76E-16 |
| IGLJ3 | 0.17084681 | 0.57518321 | 1.75131823 | 2.17E-10 | 3.64E-10 |
| IGLV1-40 | 107.358753 | 376.995262 | 1.81210658 | 1.16E-13 | 2.27E-13 |
| IGLV1-44 | 69.488726 | 213.289004 | 1.61795876 | 2.57E-12 | 4.73E-12 |
| IGLV1-47 | 59.2526211 | 181.227131 | 1.61284808 | 2.67E-13 | 5.13E-13 |
| IGLV1-50 | 1.00816023 | 2.92588714 | 1.53714917 | 1.06E-10 | 1.80E-10 |
| IGLV1-51 | 96.0572047 | 277.315917 | 1.52956469 | 6.33E-12 | 1.14E-11 |
| IGLV2-11 | 74.5176245 | 193.855713 | 1.37932967 | 1.13E-08 | 1.75E-08 |
| IGLV2-14 | 102.787237 | 395.690481 | 1.94471122 | 1.28E-13 | 2.50E-13 |
| IGLV2-18 | 5.74974938 | 18.9200674 | 1.71834625 | 1.06E-06 | 1.50E-06 |
| IGLV2-23 | 78.0519489 | 280.646772 | 1.8462489 | 1.08E-11 | 1.92E-11 |
| IGLV2-33 | 0.28593229 | 0.98017957 | 1.77737252 | 1.71E-09 | 2.74E-09 |
| IGLV2-8 | 23.4209434 | 82.607621 | 1.8184757 | 3.32E-09 | 5.28E-09 |
| IGLV3-1 | 41.226694 | 167.513508 | 2.02262675 | 7.42E-14 | 1.47E-13 |
| IGLV3-10 | 48.8856417 | 134.379163 | 1.45882676 | 2.58E-08 | 3.93E-08 |
| IGLV3-12 | 0.70763407 | 3.23637825 | 2.19330481 | 5.46E-11 | 9.43E-11 |
| IGLV3-16 | 1.8106179 | 5.18929834 | 1.51905736 | 8.49E-10 | 1.38E-09 |
| IGLV3-19 | 85.218628 | 349.034311 | 2.03412813 | 5.99E-14 | 1.19E-13 |
| IGLV3-21 | 92.2718821 | 355.562244 | 1.94613915 | 7.42E-16 | 1.60E-15 |
| IGLV3-25 | 110.255414 | 285.791776 | 1.37411491 | 5.96E-13 | 1.13E-12 |
| IGLV3-27 | 12.7708032 | 38.8447151 | 1.60486906 | 7.84E-06 | 1.06E-05 |
| IGLV3-32 | 0.10615655 | 0.41562448 | 1.96908722 | 7.50E-11 | 1.29E-10 |
| IGLV3-9 | 12.5944587 | 51.6244806 | 2.03526624 | 2.45E-14 | 4.95E-14 |
| IGLV4-3 | 0.9416688 | 4.55519869 | 2.27422234 | 0.00021159 | 0.00026594 |
| IGLV4-60 | 7.1141787 | 25.1173848 | 1.81991714 | 6.73E-06 | 9.16E-06 |
| IGLV4-69 | 23.0503999 | 98.6252025 | 2.09716458 | 1.59E-11 | 2.81E-11 |
| IGLV5-37 | 1.90013693 | 6.91841538 | 1.86433825 | 4.22E-06 | 5.81E-06 |
| IGLV5-45 | 7.977039 | 32.5828127 | 2.03018591 | 1.17E-06 | 1.66E-06 |
| IGLV5-48 | 0.4475373 | 1.5992281 | 1.83729588 | 3.48E-07 | 5.03E-07 |
| IGLV5-52 | 0.23004516 | 0.72426707 | 1.65460467 | 3.11E-07 | 4.50E-07 |
| IGLV6-57 | 41.0275122 | 122.759798 | 1.5811746 | 1.77E-13 | 3.43E-13 |
| IGLV7-43 | 9.81475467 | 36.3632873 | 1.88945852 | 2.33E-08 | 3.57E-08 |
| IGLV7-46 | 12.3057882 | 42.0689051 | 1.77341721 | 1.52E-09 | 2.45E-09 |
| IGLV8-61 | 22.4220521 | 76.5040117 | 1.77061708 | 7.52E-06 | 1.02E-05 |
| IGLV9-49 | 6.58598184 | 33.5137602 | 2.34728313 | 1.21E-06 | 1.71E-06 |
| CMA1 | 0.83683535 | 0.21453373 | -1.9637393 | 5.23E-27 | 1.86E-26 |
| CYR61 | 219.855461 | 66.4327755 | -1.7265883 | 1.13E-39 | 7.89E-39 |
| EDN1 | 45.6669626 | 12.2298579 | -1.9007432 | 7.22E-43 | 6.18E-42 |
| EDN2 | 1.33739122 | 5.56248203 | 2.05630722 | 8.41E-13 | 1.58E-12 |
| FGF10 | 1.00150644 | 0.21795143 | -2.2000931 | 6.83E-57 | 2.25E-55 |
| PROK2 | 1.02151087 | 0.22985833 | -2.1518877 | 5.20E-28 | 1.93E-27 |
| SAA1 | 9.06053959 | 32.2272237 | 1.83061103 | 2.49E-07 | 3.62E-07 |
| SAA2 | 1.54042835 | 3.72610937 | 1.27433844 | 3.20E-06 | 4.43E-06 |
| SEMA3A | 0.85549003 | 2.31128518 | 1.43387234 | 0.0007908 | 0.0009628 |
| SEMA3B | 17.5975616 | 4.87895004 | -1.8507329 | 4.01E-48 | 5.05E-47 |
| SEMA3G | 14.0850593 | 1.58802012 | -3.1488645 | 8.38E-60 | 5.08E-58 |
| SEMA4B | 9.79071555 | 42.2789628 | 2.11045378 | 2.68E-48 | 3.42E-47 |
| SEMA4C | 6.06389779 | 12.4496854 | 1.03779195 | 4.82E-33 | 2.31E-32 |
| SEMA5A | 10.1248004 | 2.45095074 | -2.0464801 | 4.16E-50 | 6.19E-49 |
| SEMA5B | 0.31734806 | 0.73264742 | 1.20705306 | 4.56E-05 | 5.93E-05 |
| SEMA6A | 4.58511447 | 1.80387309 | -1.3458599 | 5.24E-35 | 2.78E-34 |
| SLIT2 | 13.2005094 | 2.47941692 | -2.4125208 | 6.50E-59 | 3.18E-57 |
| TNC | 13.9018013 | 52.1428352 | 1.9071972 | 5.31E-12 | 9.62E-12 |
| C5AR1 | 34.7950471 | 8.66123616 | -2.0062371 | 3.02E-57 | 1.06E-55 |
| CCRL2 | 7.04785524 | 2.07238716 | -1.7658907 | 3.90E-55 | 9.76E-54 |
| CMKLR1 | 7.09339475 | 3.41379857 | -1.0550983 | 1.38E-30 | 5.86E-30 |
| CX3CR1 | 4.52798877 | 1.03054395 | -2.1354643 | 2.42E-43 | 2.13E-42 |
| EDNRB | 33.6272241 | 2.67737442 | -3.6507389 | 4.17E-64 | 3.53E-61 |
| FPR1 | 21.537828 | 5.37635832 | -2.0021716 | 6.53E-43 | 5.61E-42 |
| FPR2 | 6.7933882 | 0.83192887 | -3.0295992 | 1.83E-55 | 4.84E-54 |
| GPR17 | 1.02491161 | 0.20051732 | -2.3537007 | 1.66E-49 | 2.35E-48 |
| LTB4R2 | 0.43802162 | 1.36522345 | 1.64006311 | 1.25E-21 | 3.50E-21 |
| PLXNB3 | 0.3224143 | 3.4068176 | 3.40143705 | 1.64E-47 | 1.96E-46 |
| ROBO2 | 1.48706808 | 0.55163132 | -1.4306944 | 4.31E-39 | 2.90E-38 |
| ADM | 9.13879641 | 23.556397 | 1.36604281 | 5.40E-07 | 7.75E-07 |
| ADM2 | 0.40496916 | 3.92465973 | 3.27668363 | 3.80E-57 | 1.31E-55 |
| AGRP | 4.79550291 | 0.46141 | -3.3775609 | 1.30E-59 | 7.48E-58 |
| AMH | 0.14053127 | 0.74130898 | 2.39918381 | 1.91E-07 | 2.80E-07 |
| ANGPTL7 | 1.39506914 | 0.12377529 | -3.4945414 | 2.20E-58 | 9.49E-57 |
| APLN | 17.2361283 | 3.63427523 | -2.2456962 | 1.46E-33 | 7.18E-33 |
| ARTN | 0.16588755 | 4.62004033 | 4.79962796 | 1.76E-45 | 1.82E-44 |
| BDNF | 1.29163318 | 0.42835261 | -1.5923256 | 2.33E-38 | 1.49E-37 |
| BMP2 | 22.6017067 | 6.20665978 | -1.8645427 | 5.07E-47 | 5.86E-46 |
| BMP5 | 7.39737663 | 2.93996344 | -1.3312155 | 1.93E-36 | 1.11E-35 |
| BMP7 | 0.81106708 | 9.05109701 | 3.48019952 | 1.43E-05 | 1.92E-05 |
| BMP8A | 0.19171566 | 0.85968977 | 2.16484792 | 2.66E-38 | 1.69E-37 |
| BMP8B | 0.73061043 | 1.7479246 | 1.25846869 | 2.74E-22 | 7.90E-22 |
| CALCB | 0.01784466 | 0.29923933 | 4.06773514 | 4.20E-08 | 6.35E-08 |
| CAT | 94.9267469 | 26.1816666 | -1.8582577 | 9.03E-64 | 4.70E-61 |
| CD70 | 0.3900808 | 1.5791475 | 2.01730102 | 4.49E-06 | 6.17E-06 |
| CGA | 0.0089362 | 3.53054109 | 8.6260127 | 4.79E-08 | 7.22E-08 |
| CGB7 | 0.04413903 | 0.23231044 | 2.39592723 | 5.46E-25 | 1.78E-24 |
| CHGB | 0.13640284 | 6.82054589 | 5.64394165 | 3.45E-11 | 6.01E-11 |
| CMTM2 | 1.64137917 | 0.37977481 | -2.1116924 | 9.06E-46 | 9.52E-45 |
| CORT | 0.10045161 | 0.23030593 | 1.19705083 | 3.96E-20 | 1.03E-19 |
| CSF3 | 33.505394 | 1.65029832 | -4.3435946 | 4.64E-35 | 2.47E-34 |
| CTGF | 224.984533 | 77.2479131 | -1.542258 | 8.36E-40 | 5.87E-39 |
| DKK1 | 1.67503517 | 8.61247554 | 2.36223659 | 0.00366199 | 0.00429253 |
| EGF | 0.35369617 | 1.41733033 | 2.00259355 | 6.62E-14 | 1.31E-13 |
| FAM3C | 8.46327443 | 22.2090905 | 1.39186246 | 7.92E-43 | 6.76E-42 |
| FGF11 | 0.02741918 | 0.42003855 | 3.93726428 | 3.83E-51 | 6.30E-50 |
| FGF14 | 0.5797886 | 0.23931814 | -1.2765972 | 7.54E-34 | 3.75E-33 |
| FGF18 | 1.93056685 | 0.9205914 | -1.0683916 | 2.18E-40 | 1.59E-39 |
| FGF19 | 0.00370879 | 2.44806136 | 9.36647418 | 3.44E-28 | 1.29E-27 |
| FGF7 | 3.47290127 | 1.69843268 | -1.0319374 | 1.03E-34 | 5.38E-34 |
| GAL | 0.06273581 | 3.92153184 | 5.96598444 | 3.77E-43 | 3.28E-42 |
| GAST | 0.01123864 | 1.63227369 | 7.18227231 | 6.86E-19 | 1.69E-18 |
| GDF10 | 11.3862481 | 0.96999371 | -3.5531732 | 8.05E-62 | 1.03E-59 |
| GDNF | 0.03077879 | 0.53586997 | 4.12187457 | 3.01E-32 | 1.38E-31 |
| GMFG | 40.4357338 | 13.4190891 | -1.591344 | 9.66E-56 | 2.67E-54 |
| GNRH2 | 0.09342012 | 0.47389054 | 2.34274861 | 4.57E-05 | 5.94E-05 |
| GPI | 18.8304411 | 63.8141691 | 1.76081 | 2.18E-55 | 5.72E-54 |
| GREM1 | 0.27736215 | 4.91921752 | 4.14858603 | 9.84E-50 | 1.42E-48 |
| GUCA2A | 1.06529521 | 0.32335529 | -1.7200612 | 2.23E-50 | 3.40E-49 |
| HBEGF | 48.1474342 | 12.2711722 | -1.9721858 | 1.50E-45 | 1.55E-44 |
| HDGF | 50.910337 | 121.505827 | 1.25499497 | 2.85E-56 | 8.53E-55 |
| IFNE | 0.02422236 | 0.2888482 | 3.57590042 | 1.40E-11 | 2.49E-11 |
| IL11 | 0.1589095 | 1.07414309 | 2.75690887 | 2.13E-36 | 1.23E-35 |
| IL16 | 3.75487578 | 1.81311915 | -1.0502914 | 3.86E-35 | 2.07E-34 |
| IL17C | 0.04428201 | 0.41964425 | 3.2443742 | 1.19E-09 | 1.93E-09 |
| IL17D | 1.12475958 | 0.38957353 | -1.5296491 | 2.61E-51 | 4.37E-50 |
| IL23A | 0.61688201 | 3.28476257 | 2.41272262 | 7.13E-47 | 8.15E-46 |
| IL33 | 33.9941649 | 8.83629894 | -1.943773 | 1.08E-50 | 1.71E-49 |
| IL6ST | 39.2879702 | 19.427508 | -1.0159868 | 7.16E-42 | 5.73E-41 |
| INHA | 0.08927634 | 4.37882068 | 5.61612071 | 6.92E-35 | 3.64E-34 |
| INHBE | 0.07410394 | 0.44213084 | 2.57685123 | 3.24E-19 | 8.10E-19 |
| INSL3 | 0.12899587 | 0.41617005 | 1.68984831 | 8.58E-27 | 3.02E-26 |
| INSL4 | 0.00617946 | 1.47061969 | 7.89472663 | 8.12E-06 | 1.10E-05 |
| JAG1 | 8.35068415 | 24.6183461 | 1.55976754 | 0.00650427 | 0.0075111 |
| JAG2 | 4.57840074 | 10.0141293 | 1.12912133 | 6.16E-13 | 1.16E-12 |
| KL | 3.49095761 | 0.58209028 | -2.584308 | 1.26E-58 | 5.69E-57 |
| LEFTY2 | 1.43381131 | 0.30252822 | -2.2447136 | 1.23E-48 | 1.61E-47 |
| LHB | 0.10410323 | 0.43658776 | 2.06825682 | 8.01E-27 | 2.82E-26 |
| LTBP2 | 49.4915644 | 23.1271863 | -1.0975929 | 6.98E-28 | 2.58E-27 |
| LTBP4 | 54.9038654 | 14.784496 | -1.8928227 | 3.48E-52 | 6.34E-51 |
| MDK | 16.992921 | 131.486672 | 2.95191079 | 2.03E-54 | 4.71E-53 |
| MIA | 0.0840051 | 1.0254131 | 3.60958449 | 0.00256602 | 0.00303411 |
| NDP | 0.08233775 | 0.61027703 | 2.88983828 | 3.49E-07 | 5.05E-07 |
| NMB | 6.28251572 | 14.2477066 | 1.18131543 | 1.00E-10 | 1.71E-10 |
| NPPC | 0.08808589 | 3.2218369 | 5.19282875 | 2.67E-14 | 5.38E-14 |
| NRG4 | 0.20018687 | 0.48887991 | 1.28813277 | 4.22E-05 | 5.49E-05 |
| NRTN | 0.52162697 | 1.49916976 | 1.52307337 | 2.19E-15 | 4.62E-15 |
| NTF4 | 0.76454419 | 1.55812308 | 1.0271374 | 0.00014942 | 0.00018932 |
| OGN | 10.5347799 | 1.5891816 | -2.7288043 | 1.80E-52 | 3.39E-51 |
| OXT | 0.07070607 | 0.28314415 | 2.00163079 | 5.84E-07 | 8.36E-07 |
| PDGFB | 18.0302874 | 6.96405137 | -1.3724236 | 1.03E-38 | 6.73E-38 |
| PDGFRL | 2.56651353 | 5.78519856 | 1.17255661 | 4.31E-13 | 8.21E-13 |
| PGF | 1.3963764 | 4.29980661 | 1.62258389 | 1.30E-33 | 6.41E-33 |
| PNOC | 0.39348236 | 0.97182525 | 1.30439797 | 7.41E-13 | 1.40E-12 |
| PTHLH | 0.38076192 | 29.5521505 | 6.27823009 | 6.26E-33 | 2.98E-32 |
| REG1A | 0.01690522 | 2.55928566 | 7.242129 | 1.17E-09 | 1.91E-09 |
| RETN | 27.5082999 | 2.59051532 | -3.408556 | 2.11E-58 | 9.19E-57 |
| SCG2 | 0.81031659 | 8.02742304 | 3.30837934 | 4.02E-05 | 5.25E-05 |
| SCT | 0.58771168 | 1.30455047 | 1.15037228 | 4.11E-10 | 6.80E-10 |
| SLURP1 | 0.01969239 | 0.67445511 | 5.09801221 | 7.83E-14 | 1.54E-13 |
| SPP1 | 13.9968206 | 367.764973 | 4.71561302 | 1.17E-53 | 2.50E-52 |
| STC2 | 2.30541531 | 6.48948744 | 1.49307786 | 1.11E-09 | 1.81E-09 |
| TGFA | 3.04001838 | 10.2709273 | 1.75641449 | 1.20E-30 | 5.10E-30 |
| THPO | 0.17150832 | 0.96882593 | 2.49795894 | 0.00066036 | 0.00080723 |
| TNFSF12 | 29.8208998 | 11.3570411 | -1.3927368 | 2.63E-60 | 1.85E-58 |
| TNFSF13 | 19.8719995 | 7.30778313 | -1.4432313 | 6.44E-56 | 1.83E-54 |
| TNFSF18 | 0.31027655 | 0.88899879 | 1.5186268 | 5.11E-05 | 6.62E-05 |
| UCN | 0.53107374 | 1.20701814 | 1.18446324 | 7.38E-09 | 1.15E-08 |
| UCN2 | 0.02661031 | 1.40300162 | 5.72038764 | 2.47E-41 | 1.92E-40 |
| VGF | 0.04125456 | 1.64097163 | 5.31385294 | 9.01E-36 | 4.99E-35 |
| ACVR1C | 0.11775683 | 0.49758289 | 2.07912611 | 3.16E-16 | 6.93E-16 |
| ACVRL1 | 36.89685 | 5.73285301 | -2.6861725 | 6.09E-64 | 4.13E-61 |
| ADRB1 | 10.1711305 | 1.14658343 | -3.1490668 | 9.58E-59 | 4.48E-57 |
| ADRB2 | 12.2314444 | 1.65360401 | -2.8869091 | 1.12E-63 | 5.06E-61 |
| AGTR1 | 2.32188147 | 0.52588014 | -2.1424884 | 5.35E-56 | 1.54E-54 |
| AGTR2 | 11.9889258 | 1.9129002 | -2.6478689 | 3.85E-49 | 5.27E-48 |
| ANGPT1 | 7.78063648 | 1.51854153 | -2.3572018 | 1.94E-57 | 7.06E-56 |
| ANGPT4 | 1.44033163 | 0.08102954 | -4.1518092 | 2.74E-62 | 4.38E-60 |
| ANGPTL1 | 4.00597476 | 0.68344127 | -2.5512641 | 4.08E-59 | 2.10E-57 |
| APLNR | 9.21280827 | 3.48230667 | -1.4035977 | 5.77E-22 | 1.64E-21 |
| AVPR2 | 1.22328325 | 0.28798557 | -2.08669 | 4.65E-52 | 8.35E-51 |
| BMPR2 | 20.6732868 | 8.64938611 | -1.2570981 | 3.28E-49 | 4.53E-48 |
| CALCRL | 32.8348504 | 5.41229654 | -2.6009151 | 4.59E-59 | 2.32E-57 |
| CNTFR | 1.38101894 | 0.62233968 | -1.149959 | 6.42E-37 | 3.79E-36 |
| CRIM1 | 29.4975868 | 12.6696997 | -1.2192146 | 2.82E-48 | 3.58E-47 |
| CRLF1 | 2.59288998 | 27.6129995 | 3.41271472 | 0.00053537 | 0.00065737 |
| CSF3R | 15.2946476 | 5.56465701 | -1.4586622 | 2.31E-41 | 1.80E-40 |
| EGFR | 10.5478459 | 23.7191685 | 1.16910505 | 1.98E-06 | 2.77E-06 |
| ENG | 109.36495 | 31.1643127 | -1.8111837 | 9.87E-59 | 4.59E-57 |
| FGFR4 | 16.1627415 | 2.6796401 | -2.5925608 | 6.54E-59 | 3.18E-57 |
| FLT4 | 5.98445716 | 1.89252777 | -1.6609059 | 7.50E-48 | 9.25E-47 |
| GALR2 | 0.05571643 | 0.31994357 | 2.52164275 | 1.69E-20 | 4.50E-20 |
| GCGR | 0.00866041 | 0.23205726 | 4.74390153 | 2.20E-28 | 8.35E-28 |
| HNF4G | 0.15746754 | 1.40066425 | 3.15298485 | 3.31E-35 | 1.78E-34 |
| HTR3A | 0.05596369 | 2.2499382 | 5.32925039 | 4.41E-37 | 2.63E-36 |
| HTR3C | 1.4489855 | 0.1268739 | -3.513576 | 2.33E-57 | 8.35E-56 |
| IL12RB2 | 0.1363334 | 0.70303559 | 2.36645871 | 1.04E-17 | 2.44E-17 |
| IL17RD | 0.39731316 | 1.13616 | 1.51581754 | 6.18E-22 | 1.75E-21 |
| IL18R1 | 3.46442033 | 1.38916915 | -1.3183917 | 3.73E-27 | 1.33E-26 |
| IL18RAP | 1.19436218 | 0.58612966 | -1.0269486 | 9.04E-19 | 2.21E-18 |
| IL1R2 | 1.40689469 | 3.4014629 | 1.27364101 | 1.29E-05 | 1.73E-05 |
| IL1RL1 | 10.6852421 | 1.2103726 | -3.1420965 | 1.63E-51 | 2.79E-50 |
| IL1RL2 | 0.30302821 | 1.48204216 | 2.29006247 | 1.06E-42 | 8.94E-42 |
| IL20RB | 0.30157672 | 10.3841892 | 5.1057197 | 6.62E-41 | 5.01E-40 |
| IL22RA1 | 0.59998875 | 2.0720866 | 1.78807695 | 5.63E-15 | 1.17E-14 |
| IL22RA2 | 0.04880875 | 0.51170421 | 3.39009842 | 2.24E-41 | 1.74E-40 |
| IL2RA | 1.36830295 | 3.52056458 | 1.36341912 | 1.36E-17 | 3.17E-17 |
| IL31RA | 0.04844245 | 0.7000897 | 3.85319593 | 3.46E-27 | 1.24E-26 |
| IL3RA | 20.6086653 | 5.64977227 | -1.8669865 | 8.17E-60 | 4.98E-58 |
| IL5RA | 1.34720934 | 0.28639025 | -2.2339198 | 8.55E-44 | 7.75E-43 |
| IL6R | 15.1758782 | 7.53722363 | -1.0096749 | 6.38E-35 | 3.37E-34 |
| KDR | 17.9184049 | 7.95246448 | -1.1719683 | 7.72E-36 | 4.30E-35 |
| LEPR | 6.36724792 | 1.51397009 | -2.0723332 | 4.87E-57 | 1.65E-55 |
| LGR4 | 1.74218161 | 10.5495495 | 2.59821446 | 3.08E-57 | 1.08E-55 |
| LIFR | 16.0014888 | 4.95092458 | -1.6924363 | 4.34E-47 | 5.04E-46 |
| MC1R | 0.46925553 | 1.10999818 | 1.24211165 | 7.03E-23 | 2.08E-22 |
| MCHR1 | 0.11739199 | 0.36455675 | 1.63480951 | 6.03E-23 | 1.79E-22 |
| MET | 15.9386249 | 33.3365616 | 1.06457814 | 0.00013807 | 0.00017522 |
| NPR1 | 16.3752417 | 2.09838164 | -2.9641672 | 1.33E-62 | 2.48E-60 |
| NPR3 | 6.62548245 | 3.1508108 | -1.0723024 | 4.65E-28 | 1.73E-27 |
| NR0B1 | 0.02262943 | 3.99361493 | 7.46335146 | 3.10E-05 | 4.07E-05 |
| NR2F1 | 12.904289 | 5.81909301 | -1.1489844 | 6.10E-36 | 3.41E-35 |
| NR3C2 | 4.85220308 | 1.9130175 | -1.3427899 | 1.11E-41 | 8.81E-41 |
| NR4A1 | 58.8177641 | 13.6627879 | -2.106 | 4.16E-38 | 2.62E-37 |
| NR4A2 | 16.6846355 | 6.98083777 | -1.2570481 | 2.16E-20 | 5.71E-20 |
| NR4A3 | 11.7167654 | 2.09060077 | -2.4865849 | 7.83E-37 | 4.60E-36 |
| NR5A1 | 0.00738281 | 0.3121495 | 5.40192321 | 7.98E-15 | 1.64E-14 |
| NR5A2 | 1.26194204 | 0.43985394 | -1.5205492 | 4.05E-30 | 1.68E-29 |
| PTGER4 | 10.9276314 | 4.42764301 | -1.3033699 | 9.09E-48 | 1.11E-46 |
| PTGFR | 1.58639285 | 0.65441037 | -1.2774826 | 3.08E-45 | 3.11E-44 |
| PTH1R | 3.3199879 | 0.7705958 | -2.1071318 | 4.37E-58 | 1.79E-56 |
| RARG | 5.38687091 | 12.2597357 | 1.18640848 | 2.26E-19 | 5.71E-19 |
| RXFP1 | 1.79468699 | 0.27636681 | -2.699076 | 2.75E-58 | 1.16E-56 |
| RXRG | 2.43816316 | 0.4238771 | -2.5240767 | 1.44E-55 | 3.89E-54 |
| S1PR1 | 52.3997456 | 6.6234046 | -2.9839149 | 2.93E-64 | 2.88E-61 |
| SDC1 | 93.3755148 | 248.758332 | 1.41362865 | 7.83E-33 | 3.71E-32 |
| SORT1 | 27.5755009 | 13.5571743 | -1.0243306 | 4.27E-47 | 4.96E-46 |
| SSTR1 | 4.41058415 | 0.74531981 | -2.5650382 | 2.15E-53 | 4.45E-52 |
| SSTR2 | 0.20765247 | 0.59707342 | 1.5237373 | 3.35E-06 | 4.64E-06 |
| TEK | 20.6907033 | 1.94951991 | -3.4077919 | 2.00E-64 | 2.70E-61 |
| TGFBR2 | 116.009001 | 29.7194679 | -1.9647566 | 4.75E-63 | 1.30E-60 |
| TGFBR3 | 10.7060269 | 2.42544879 | -2.1420996 | 1.29E-56 | 4.11E-55 |
| TIE1 | 15.3542079 | 3.32657101 | -2.2065264 | 5.94E-61 | 5.03E-59 |
| TNFRSF17 | 1.25300002 | 3.46775487 | 1.46861548 | 4.24E-11 | 7.36E-11 |
| TNFRSF18 | 1.11241495 | 10.3863466 | 3.22292133 | 2.77E-46 | 3.02E-45 |
| TNFRSF21 | 15.1915126 | 56.8045875 | 1.90274192 | 2.81E-49 | 3.90E-48 |
| TNFRSF25 | 1.07029968 | 4.63290568 | 2.11390251 | 2.29E-38 | 1.46E-37 |
| TNFRSF9 | 0.54580203 | 1.21152336 | 1.15037255 | 4.75E-12 | 8.64E-12 |
| TUBB3 | 0.08688623 | 1.64142253 | 4.23967531 | 3.22E-55 | 8.18E-54 |
| VIPR1 | 18.1626005 | 1.95184603 | -3.2180596 | 1.62E-55 | 4.32E-54 |
| ICAM2 | 9.68004318 | 2.9190091 | -1.7295348 | 8.21E-57 | 2.68E-55 |
| ITGAL | 14.1729518 | 5.54289077 | -1.3544298 | 5.58E-43 | 4.82E-42 |
| PAK1 | 6.06404696 | 18.8275081 | 1.63448923 | 3.05E-59 | 1.63E-57 |
| TYROBP | 225.316017 | 75.9965294 | -1.5679444 | 1.13E-49 | 1.63E-48 |
| FCGR3A | 83.8458618 | 33.0862267 | -1.3415088 | 3.47E-40 | 2.50E-39 |
| FCGR3B | 4.9410175 | 1.2218505 | -2.0157404 | 2.71E-33 | 1.31E-32 |
| NCR1 | 0.43288148 | 0.21519515 | -1.0083265 | 1.84E-27 | 6.67E-27 |
| SHC3 | 2.96641936 | 0.72978522 | -2.0231787 | 1.10E-51 | 1.91E-50 |
| HCST | 18.761538 | 8.52847542 | -1.1374183 | 3.86E-37 | 2.31E-36 |
| CD244 | 1.79172597 | 0.70043388 | -1.3550292 | 2.55E-35 | 1.38E-34 |
| SH2D1B | 0.93646891 | 0.26333815 | -1.8303145 | 1.12E-40 | 8.34E-40 |
| PRF1 | 16.2382177 | 6.42049222 | -1.3386375 | 3.11E-34 | 1.58E-33 |
| MAP3K8 | 12.215124 | 5.39206524 | -1.1797587 | 5.63E-35 | 2.98E-34 |
| CTLA4 | 1.00202549 | 2.05193127 | 1.03406319 | 1.11E-10 | 1.88E-10 |
| CBLC | 1.35013054 | 18.5802632 | 3.78260013 | 6.16E-57 | 2.04E-55 |
| CDK4 | 13.6952126 | 36.5500641 | 1.41620227 | 4.84E-48 | 6.06E-47 |
| PDK1 | 0.77272532 | 3.3259484 | 2.10573821 | 1.47E-54 | 3.46E-53 |
| PRKCQ | 4.30029055 | 1.6191972 | -1.4091554 | 3.85E-48 | 4.86E-47 |
| TRGJP2 | 5.82057718 | 1.25562546 | -2.212756 | 5.35E-45 | 5.29E-44 |
| TRGC2 | 2.40264324 | 1.04373208 | -1.202871 | 4.81E-32 | 2.19E-31 |
| TRGC1 | 0.63865694 | 0.27489643 | -1.216153 | 3.70E-40 | 2.67E-39 |

NOTE: Control: mean gene expression of adjacent non-tumor tissues; Tumor: mean gene expression of tumor tissues; FC: fold change; *P*-value: *P*-value from Wilcoxon rank-sum test; FDR: false discovery rate.
